# Clinician views concerning the prevalence and impact of granulomas on the diagnosis, management, and outcomes of ANCA-associated vasculitis

**DOI:** 10.1093/rheumatology/keaf585

**Published:** 2026-02-04

**Authors:** Mats Junek, Lynn A. Fussner, Arielle Mendel, David Jayne, Peter A. Merkel, Michael Walsh

**Affiliations:** 1 Division of Rheumatology, McMaster University, Hamilton, ON, Canada; 2 Division of Health Research Methods, Evidence, and Impact, McMaster University, Hamilton, ON, Canada; 3 Division of Pulmonary, Critical Care, and Sleep Medicine, The Ohio State University Wexner Medical Center, Columbus, OH, USA; 4 Division of Rheumatology, McGill University Health Centre, Montreal, QC, Canada; 5 Department of Medicine, University of Cambridge, UK; 6 Division of Rheumatology, Department of Medicine, Division of Epidemiology, Department of Biostatistics, Epidemiology, and Informatics, University of Pennsylvania, Philadelphia, PA, USA; 7 Department of Medicine, McMaster University, Hamilton, ON, Canada

**Keywords:** anti-neutrophil cytoplasm antibody, vasculitis, histopathology, qualitative, attitude of health professionals

## Abstract

**Objectives:**

It is unclear whether clinicians agree which manifestations of ANCA-associated vasculitides (AAV) is associated with necrotizing granulomas or if their presence affects clinical decision-making.

**Methods:**

We surveyed physicians experienced in caring for individuals with AAV, querying: experience with AAV; beliefs concerning how granulomas affect the diagnosis, treatments and outcomes of AAV; beliefs concerning the frequency with which granulomas are found in 36 manifestations of AAV; and degree to which granulomas change choice of induction therapy for specific manifestations of AAV. We analysed responses using descriptive statistics and multivariable linear regression.

**Results:**

We received 142 responses from 35 countries. Responses had a median Likert response ≥5 on a seven-point scale (equal to ‘partially agree’) that granulomatous manifestations respond differently to therapy, increase risk of relapse and increase organ damage. Four of 36 manifestations were believed to be caused by granulomas in a median of ≥ 75% of cases (on a scale of 0 = never to 100 = always caused by granuloma), 19 in a median of ≤25% of cases, and 13 in intermediate medians. The perceived degree to which granulomas caused manifestations was not associated with changes in therapy to induce remission in severe AAV (*P*-values 0.26–0.93 across scenarios).

**Conclusions:**

Physicians experienced in vasculitis generally agree on which manifestations of AAV are and are not caused by granulomas and that granulomatous inflammation alters the natural history and treatment of AAV. However, the presence of granulomatous manifestations did not alter treatment choices to induce remission in severe AAV.

## Introduction

The ANCA-associated vasculitides (AAV) are a group of relapsing, potentially life-threatening multisystem diseases characterized by tissue damage associated with vasculitis and/or necrotizing granulomatous inflammation. Vasculitic manifestations in AAV are typically defined by the presence of capillaritis in the absence of granulomas whereas granulomatous manifestations demonstrate necrotizing granulomas that are often accompanied by vasculitis [1, 2]. These two pathological findings are present in varying proportions across organs and may contribute to different disease courses [3–5]. Limited data have characterized some manifestations as predominantly due to necrotizing granulomas. However, the degree to which health care providers agree on which manifestations are due to granulomatous inflammation, as well as the effect on treatment decisions and outcomes of patients with AAV, is uncertain [6–10].

Consensus over which manifestations of AAV are caused by necrotizing granulomas may aid the study and management of AAV. The term ‘limited’ AAV has historically been used to sometimes label individuals with predominantly granulomatous manifestations (e.g. isolated sinonasal or orbital) and was intended to distinguish a disease phenotype that may not be immediately organ- or life-threatening (i.e. ‘severe’). This categorization may be applied based on individual or cumulative features, and factored into the intensity of the initial approach to treatment. Associating granulomatous manifestations with ‘non-severe’ disease may, however, misrepresent the importance of such features on health-related quality of life, disease-related damage and link to frequency of relapse [11–15]. In addition to these patient factors, dividing manifestations into either granulomatous or non-granulomatous creates a false dichotomy as granulomas or vasculitis (or both) may be demonstrated in the histopathology for the same manifestation in different patients, such as in cardiac, lung and cutaneous findings [4–6, 16–18]. Categorizing the specific histopathology of lesions in AAV may also influence treatment based the mechanism of action of available drugs for vasculitis.

Better consensus is needed to define granulomatous manifestations of AAV and how they impact the disease. We developed and delivered an international survey of experts in vasculitis to improve our understanding of what are clinician perceptions of granulomatous manifestations of AAV and how these perceptions may change the care of individuals with these manifestations.

## Methods

We developed and conducted an international survey of physicians who care for individuals with AAV to understand the degree to which physicians believe granulomas affect the diagnosis, treatments and outcomes of AAV; the frequency with which granulomas cause manifestations of AAV; and scenarios that assessed respondents’ induction treatment for severe AAV with different manifestations varying in the degree to which they are believed to be caused by granulomatous inflammation. The term granuloma was used to encompass the spectrum of granulomatous inflammation described in AAV [1, 2]. We carried out the survey in two phases: a development phase that generated the items assessing beliefs concerning granulomas assessed with a limited group of physician respondents, and a deployment phase that included other aspects of granulomas, which was administered across a wider audience. This study protocol received ethics approval from the local research ethics board (Hamilton Integrated Research Ethics Board, approval 16609). No individuals with AAV took part in this study.

### Survey development and validation

We first created a set of items to assess respondents’ beliefs regarding the degree to which granulomas affect (i) the pathobiology, presentation, and diagnosis of AAV; (ii) how clinicians engage with patients concerning AAV; (iii) the treatment of AAV; and (iv) prognostication and outcomes of AAV succinctly while maintaining content validity. We generated a candidate pool of 29 items structured as statements concerning the beliefs regarding granulomas on an aspect of that domain (e.g. ‘granulomatous manifestations of AAV have a different pathobiology’). We chose a seven-point Likert scale as the response format. These items underwent evaluation by two experienced vasculitis clinician–researchers who assessed the items for content validity, redundancy, semantic ambiguity and feasibility of administration, which resulted in a final pool of 24 items that were used as a developmental survey for further evaluation. We solicited 23 vasculitis experts in Canada, the United States and the United Kingdom who were known to the authors to complete this survey for development. Twelve of the 23 clinicians with experience caring for patients with vasculitis who were approached (52.2%) completed the preliminary survey. Items from the developmental survey were removed: if they had item-total correlations ≤0.30, if they could be removed without compromising face validity, if they exhibited floor or ceiling effects (defined as mean response ≥5.5 or ≤2.5 with range ≤2), and/or if they had a load of ≤0.4 across all factors using a varimax rotated factor analysis [19]. They also provided feedback concerning the form and content of the survey. With this information we reduced the beliefs concerning granulomas item set to 14 while preserving content validity and demonstrated acceptable psychometric performance.

We then added three item sets to the beliefs concerning granulomas for a total of four sets. The first set (preceding the beliefs concerning granulomas) assessed respondent demographics including their country, specialty, how long they had been in practice and how many individuals with AAV they have cared for, the extent to which they provide collaborative care, and time spent doing research. The second added set consisted of 36 items, each of which asked the frequency with which granulomas caused a manifestation of AAV using the manifestations listed in the Birmingham Vasculitis Activity Score version 3 (BVASv3), the most commonly used tool for cataloguing manifestations of disease in clinical trials in AAV [20]. The response format was a slider anchored at ‘Never caused by granulomas’ (0) to ‘Always caused by granulomas’ (100) with a midway marker ‘Caused by granulomas in 50% of patients’.

The final added item set consisted of four scenarios of patients with severe AAV, with the most severe manifestation being one that is, in the literature, histologically associated with necrotizing granulomatous inflammation (nodular lung lesions, retro-orbital tumour, conductive hearing loss, or pachymeningitis). The respondent was asked what therapy for induction of remission they would choose in addition to oral glucocorticoids, the rationale for this therapy, and the degree to which they agreed that they would change therapy if the most severe manifestation changed to one in the same organ system but not conventionally associated with granulomas (diffuse alveolar haemorrhage, scleritis, sensorineural hearing loss, and mononeuritis multiplex respectively). The response format was a seven-point Likert scale anchored around ‘neither agree nor disagree’. To reduce survey fatigue, items were ordered randomly and each respondent was only asked to respond to two of the four scenarios at random. The final survey can be found in Supplementary Data S1.

### Survey administration

We uploaded the survey questions and organized them for administration using an internet-based survey service (www.surveymonkey.com). The survey was sent to individuals on mailing lists of four vasculitis research societies: the Canadian Vasculitis Research Network, the Vasculitis Clinical Research Consortium, the European Vasculitis Society, and the Australia and New Zealand Vasculitis Society. These mailing lists collectively consist of over 800 addresses; however, due to individuals being on multiple mailing lists, we were unable to determine the total number of potential respondents. The survey was opened on 27 October 2023 and closed on 29 February 2024.

### Analysis

Individuals were excluded from analysis if they did not consent, indicated that they were repeating the survey, or only provided demographic information and did not respond to any other items. Data for each item set was summarized using number, frequency, median and interquartile range (IQR) as appropriate. Based on an initial review of the data, manifestations of AAV underwent *post hoc* categorization: those with a median ≥75% (scale 0% to100%) across all respondents were considered granulomatous, those with a score ≥50% and <75% were considered frequently granulomatous, ≥25% to <50% infrequently granulomatous, and <25% not granulomatous.

We used the responses from the patient scenarios to then assess how much an individual’s beliefs concerning granulomas may impact therapeutic decision-making. Mixed-effects, multivariable linear regression was performed for responses of each scenario where respondents were treated as random intercepts; the outcome was the respondent’s Likert response as to whether a change in manifestation would lead to a change in therapy. The scenario, respondent’s duration in practice, number of individuals with vasculitis that they care for, how frequently the respondent believed the first and second manifestations were caused by granulomas, and frequency with which the respondent cared for individuals with AAV with another specialty were fixed effects. All analysis was performed using SAS Viya version 3.5 (SAS Institute, Cary, NC, USA) [21].

## Results

Of 161 respondents, one did not consent, four were repeat responses, and 14 did not provide any granuloma-related responses. Therefore, data from 142 respondents from 35 countries were included for analysis. Most respondents were from Europe or North America, and responses were distributed across a variety of levels of clinical experience, research experience and care for patients in collaborative clinics (Table 1). Ninety-four (66.2%) responses were from rheumatologists, 31 (21.8%) from nephrologists, nine (6.3%) from internal medicine and eight (5.6%) other specialists.

### Beliefs concerning the impact of granulomas on AAV diagnosis, management, and outcomes

Responses indicated there was median Likert response of 6 (agree) that granulomas were associated with an increased risk of relapse and median response of 1 (strongly disagree) that granulomas were necessary for a diagnosis of microscopic polyangiitis. There was a median response of 5 (somewhat agree) that granulomatous manifestations of AAV had a differential response to therapy, required higher cumulative doses of glucocorticoids, had increased risk of relapse, and that patients with granulomatous manifestations developed more permanent damage than those who did not have such manifestations. Full responses and histograms are seen in Supplementary Table S1. The item with the greatest variability was ‘The presence of symptomatic granulomas is necessary for granulomatosis with polyangiitis’.

### Granulomatous manifestations of AAV

**Figure 1.**
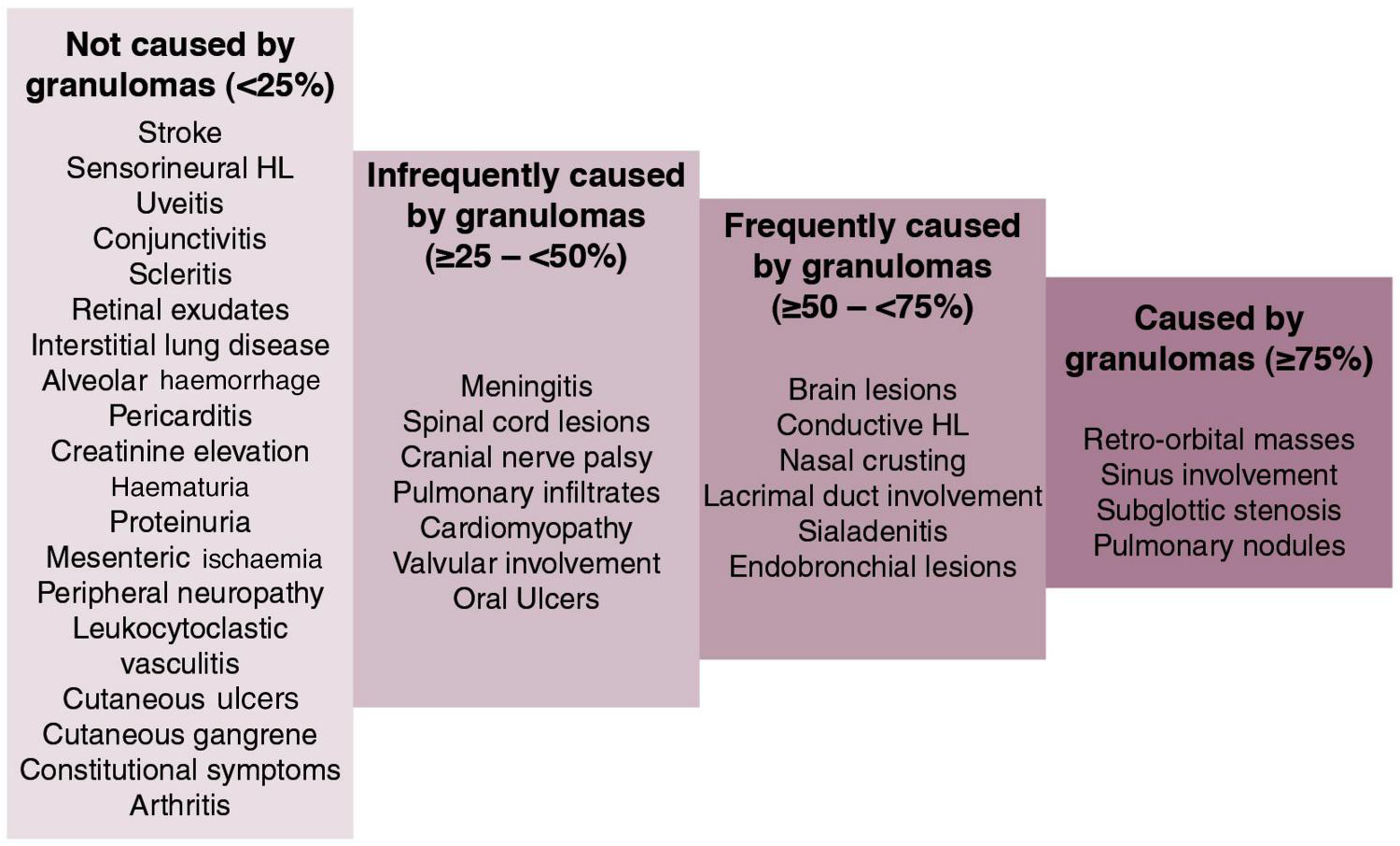
Manifestations of ANCA-associated vasculitis grouped by the median frequency (percentages) that respondents answered that a given manifestation was caused by granulomas. HL: hearing loss

When asked if each of 36 manifestations of AAV was caused by granulomas (where 0 is never, 100 is always), four items were considered granulomatous (11.1% with median score of ≥ 75%), six were frequently granulomatous (median score ≥50% and <75%), seven infrequently granulomatous (≥25% and <50%), and 19 (52.7%) were considered to not be granulomatous (median scores <25%) (Fig. 1). The manifestation believed to be most frequently caused by granulomas was pulmonary nodules (median 88%, IQR 72–99) and least frequently caused by granulomas was arthritis (3.5%, IQR 0–16). Data for responses to each manifestation can be seen in Supplementary Table S2.

### Influence of granulomatous manifestations of AAV on treatment decisions

Respondents completed 230 of the 284 scenarios (81.0%). The most common therapeutic regimens for induction of remission for individuals with nodular lung disease, sensorineural hearing loss, or pachymeningitis was glucocorticoids with rituximab (63.6–72.7% of respondents) (Table 2). The most common therapeutic regimen for induction of remission for retro-orbital pseudotumour was glucocorticoid monotherapy (49.2%) followed by rituximab (35.6%). Respondents changed their therapeutic decision in 34.5–70.4% of scenarios. They were least likely to change when switched from conductive to sensorineural hearing loss, and were most likely to switch when retro-orbital pseudotumour was switched to scleritis.

Respondents had a median Likert response of 5 (partial agreement) that their choice of therapy was consistent with previous treatments, was the local therapeutic norm, treated the underlying pathobiology, and was an evidence-based therapy (Supplementary Table S3). There was a median response of 3 (somewhat disagreement) that respondents would change therapy if the new manifestation was diffuse alveolar haemorrhage (originally nodular lung disease) or mononeuritis multiplex (originally pachymeningitis); and the median response was 4 (neither agreement nor disagreement) with changing therapy for scleritis (originally retro-orbital pseudotumor) or sensorineural hearing loss (originally conductive hearing loss).

Results of the multivariable linear regression assessing the association between the respondent’s beliefs surrounding granulomas and likelihood to change therapy demonstrated that the frequency with which individuals believed that a given manifestation of AAV was caused by granulomas was not associated with a change in choice of treatment (Table 3). This did not change when the regression was repeated with each scenario individually (not shown). A longer duration in practice was associated with a lower likelihood that the respondent would agree that they would change induction therapies (*P* < 0.01).

## Discussion

Our survey demonstrates that clinicians believe granulomatous manifestations of AAV have differential responses to therapy and outcomes; respondents agreed that 75% of manifestations of AAV can be categorized as either caused by or not caused by granulomas. These differences do not, however, appear to factor into decision-making when prescribing therapy for induction of remission for severe disease.

Sinus and other otolaryngological manifestations of AAV formed the majority of the manifestations that respondents believed to be caused by granulomas in patients with AAV. These manifestations are frequently seen in individuals with granulomatosis with polyangiitis and have been associated with increased damage and relapse, as well as the presence of necrotizing granulomatous inflammation [4, 11, 22, 23]. Histologically identified granulomas are, however, found in manifestations that our respondents did not believe granulomatous, such as scleritis and cutaneous ulcers [16, 24–26]. Reports with pathological findings may be subject to selection bias and feature cases considered to be atypical, and it is important to note that not all features of AAV are easily amenable to biopsy, which may create information bias. It is also possible that clinicians derived their beliefs concerning granulomas based on the type of AAV in which the manifestation occurs (either from experience or from reported associations) or an associated disease course [1]. Clarifying the definition of granulomatous disease, including the degree to which necrotizing granulomas are thought to be causative of the changes (*vs* being seen in the tissue and the location/manifestation itself causing the altered natural history) may have helped to separate if they are considered a clinical entity, pathologic entity, or both.

We found differences in the approach to treatment to induce remission based on individual manifestations of AAV; however, we did not find that respondents intended to change their therapy based on the extent to which an individual believed that manifestations were caused by granulomas. Respondents instead indicated that changes in treatment reflected local practice norms, effectiveness, and that the therapy treated the underlying pathobiology of AAV. We also saw that not all manifestations associated with granulomas similarly impacted decisions: retro-orbital pseudotumours were treated with rituximab with glucocorticoids *vs* glucocorticoid monotherapy in 47.7% and 34.1% of cases, respectively, and lung nodules in 74.1% and 1.9% of cases, respectively. Factors that appeared to drive choice of initial therapy for these severe manifestations included availability, familiarity, and previous experience with the treatments. The scenarios in which change most frequently (retro-orbital pseudotumor changing to scleritis) and least frequently (conductive changing to sensorineural hearing loss) occurred both had median Likert responses of neither agree nor disagree likelihood to change therapy, suggesting that treatment also has a component of the gestalt that was not addressed by survey items. The survey section assessing beliefs concerning granulomas found agreement that granulomatous manifestations required higher cumulative doses of glucocorticoids and had a differential response to therapy. This suggests that beliefs about granulomatous manifestations may be associated with other aspects of therapy for AAV not assessed in the scenarios such as the number of agents, duration of therapy and approaches to glucocorticoid tapering.

This study has several strengths, foremost that it offers an avenue to interrogate an aspect of AAV that has been of interest to the field but not previously directly engaged. We solicited responses from over 100 individuals with expertise in vasculitis from 35 countries, providing a breadth of responses that can be difficult to achieve in rare diseases, and had a high level of survey engagement with complete data. We also considered how granulomatous manifestations interact with AAV across multiple areas, opening new avenues for research into the classification, treatment and outcomes of this disease.

Several limitations should be considered when interpreting these findings. We solicited the input of clinicians who self-reported as experts in treating individuals with vasculitis but did not define expertise for participation in the survey nor verify their experience. Our results were heterogeneous, likely reflecting a breadth of experiences, expertise, knowledge and a lack of clear consensus on this subject. We also did not know the response rate, as mailing lists often had overlapping recipients, and it is unknown if respondents are a representative sample of vasculitis clinicians. In designing the survey, we sought to assess how clinician beliefs concerning granulomatous manifestations may affect their behaviour but did not assess their knowledge of histopathology. While this approach does not assess if granulomas are causative of a given outcome, clinical decisions are made based on clinician beliefs. Similar limitations were seen in the scenarios that focused on therapy to induce remission for severe disease rather than non-severe disease, duration of therapy or other aspects.

The questions in the scenarios also assessed different aspects of treatment for granulomatous manifestations of AAV than the section assessing general beliefs concerning granulomatous manifestations, and were not able to be used to internally validate findings. Choices of therapy for life/organ-threatening AAV is limited and has been considered equivocal in clinical trials, which may have led to the lack of difference in respondent choices, based on the overall/cumulative severity of disease beyond the initial manifestation in question. Finally, in assessing the effects of granulomas on choice of therapy, we categorized several manifestations to be either granulomatous or non-granulomatous *a priori*, whereas respondents did not agree with such a clear pathological delineation (e.g. conductive and sensorineural hearing loss). The consistency of responses across these manifestations, however, reinforces the validity of these results that changes in therapy were independent of beliefs concerning granulomas.

Our survey results suggest that physicians who treat AAV generally agree which manifestations are or are not caused by granulomas and that individuals with granulomatous manifestations of AAV differ clinically from those without them in both treatment and disease outcomes. There is, however, less consensus on how this changes therapeutic decision-making. Additionally, given that some manifestations of AAV involve a mix of granulomatous and non-granulomatous pathology, there is further complexity that this dichotomous classification cannot address. Future work should seek to reconcile these clinical views with histopathological data and assess whether granulomatous manifestations have different clinical outcomes in larger data sets. These definitions can be used to better understand disease phenotypes, choice and development of treatments, outcomes, and improve care for individuals with AAV.

## Supplementary Material

supplement 1

supplement 2

Supplementary material is available at *Rheumatology* online.

## Figures and Tables

**Table 1.** Demographics of survey respondents

Demographic	*n* (%)
Number of patients with vasculitis cared for	
<50	21 (14.8)
50–99	37 (26.1)
100–249	43 (32.3)
250–499	27 (19.0)
500+	14 (9.9)
Duration in practice	
Trainee	2 (1.4)
<5 years	9 (6.3)
5 to <10 years	26 (18.3)
10 to <15 years	31 (21.8)
15 to <20 years	22 (15.5)
20+ years	52 (36.6)
Frequency of collaboration	
0%	14 (9.9)
1–25%	36 (25.4)
26–50%	57 (40.1)
51–75%	20 (14.1)
76–100%	15 (10.6)
Time spent doing research	
0–25%	71 (50.0)
26–50%	51 (35.9)
51–75%	15 (10.6)
76–100%	5 (3.5)
Geographic area of practice	
Asia	13 (9.2)
Australasia	6 (4.3)
Europe	65 (45.8)
North America	49 (34.5)
South America	8 (5.6)
Missing	1 (0.7)

**Table 2.** Respondent choice of therapy for induction of remission for severe manifestations of ANCA-associated vasculitis within the same organ across the four proposed patient case studies

	*n*	CYC	RTX	CYC or RTX	GCs alone	Other
Nodular lung disease 88% (72–99) from granulomas	54	7 (13)	40 (74.1)	1 (1.9)	1 (1.9)	5 (9.3)
Diffuse alveolar haemorrhage 5.5% (0–20) from granulomas	54	20 (37)	29 (53.7)	2 (3.7)	1 (1.9)	2 (3.7)
Conductive hearing loss 50% (20–75) from granulomas	55	35 (63.6)	1 (1.8)	0 0	0 0	15 (27.3)
Sensorineural hearing loss 20% (5–50) from granulomas	55	0 0	0 0	11 (20)	0 0	11 (20)
Pachymeningitis 47% (15–72) from granulomas	62	19 (30.6)	39 (62.9)	3 (4.8)	1 (1.6)	0 0
Mononeuritis multiplex 15% (2.5–23.5) from granulomas	62	16 (25.8)	42 (67.7)	2 (3.2)	0 0	2 (3.2)
Retro-orbital pseudotumour 84% (60–96) from granulomas	44	3 (6.8)	21 (47.7)	1 (2.3)	15 (34.1)	4 (9.1)
Scleritis 17% (6–49) from granulomas	44	12 (27.3)	15 (34.1)	1 (2.3)	1 (2.3)	15 (34.1)

Frequency that a manifestation was considered caused by granulomas is expressed as the median and interquartile range. Responses are expressed as frequency and percent. CYC: cyclophosphamide; GCs: glucocorticoids; RTX: rituximab.

**Table 3.** Results of multivariable linear regression indicating if one would change induction therapies based on severe granulomatous *vs* vasculitic manifestations of AAV

Variable	Parameter coefficient	*P*
Intercept	5.15	<0.01
Frequency of caring for individuals with ANCA-associated vasculitis with another specialty	0.10	0.41
Duration in practice	−0.30	<0.01
Number of individuals with vasculitis cared for	−0.07	0.59
Frequency that the first manifestation was thought to be caused by granulomas	−0.003	0.49
Frequency that the second manifestation was thought to be caused by granulomas	−0.002	0.7

## Data Availability

The data underlying this article will be shared on reasonable request to the corresponding author.

## Rheumatology key messages

- We have generated a consensus list of granulomatous manifestations of AAV.
- Clinical perceptions of granulomas did not affect decision-making around treatment to induce remission in severe AAV.
- This candidate list of granulomatous manifestations can be used to guide future research pairing histology and clinical findings.
