# Supplementary material for: Clinician views concerning the prevalence and impact of granulomas on the diagnosis, management, and outcomes of ANCA-associated vasculitis
Source: Rheumatology (Oxford). Author manuscript; Available in PMC 2026 May 15. (PMC13179117; doi:10.1093/rheumatology/keaf585)
Supplement: supplement 1 [file NIHMS2174520-supplement-supplement_1.docx]

**Supplementary Data**

**Supplementary Table S1:** Responses to the beliefs concerning granulomas survey items

| Item | Minimum | Maximum | Median (IQR) |
| --- | --- | --- | --- |
| Where both vasculitis and granulomas are present in a given organ/tissue affected by AAV, the pathobiology is driven by granulomas. | 1 | 7 | 4 (3-5) |
| The presence of symptomatic granulomas is necessary for granulomatosis with polyangiitis | 1 | 7 | 4 (2-6) |
| The presence of symptomatic granulomas is necessary for microscopic polyangiitis | 1 | 7 | 1 (1-2) |
| The presence of symptomatic granulomas is necessary for ANCA-negative vasculitis | 1 | 7 | 3 (2-5) |
| Granulomatous manifestations of AAV have differential responses to therapy. | 2 | 7 | 5 (5-6) |
| In a patient with a non-granulomatous manifestation of AAV causing organ and/or life-threatening disease, the presence of other granulomatous manifestations impacts my choice of induction therapy. | 1 | 7 | 4 (2-5) |
| In a patient with a non-granulomatous manifestation of AAV causing non-organ threatening, non-life threatening disease, the presence of other granulomatous manifestations impacts my choice of induction therapy. | 1 | 7 | 5 (3-6) |
| When treating a patient with AAV, the presence of granulomatous manifestations impacts my choice of maintenance therapy. | 1 | 7 | 5 (3-6) |
| When treating a patient with AAV, the presence of granulomatous manifestations impacts my duration of therapy. | 1 | 7 | 5 (3-6) |
| Patients with granulomatous manifestations of AAV are more likely to experience relapse than patients without granulomatous manifestations | 1 | 7 | 6 (5-6) |
| Patients with granulomatous manifestations of AAV are more likely to experience death than patients without granulomatous manifestations | 1 | 7 | 3 (2-4) |
| Patients with granulomatous manifestations of AAV are more likely to experience infection than patients without granulomatous manifestations | 2 | 7 | 4 (3-5) |
| Patients with granulomatous manifestations of AAV are more likely to experience damage than patients without granulomatous manifestations | 1 | 7 | 5 (5-6) |
| Patients with granulomatous manifestations of AAV require higher cumulative doses of glucocorticoids than those without granulomatous manifestations. | 1 | 7 | 5 (3-5) |

IQR = interquartile range; AAV = ANCA-associated vasculitis

**Supplementary Table S2:** Characteristics of manifestation-specific responses for the following prompt: “Across all individuals with granulomatosis with polyangiitis and microscopic polyangiitis, how frequently are the following manifestations caused by granuloma?”

| **Manifestation** | **Minimum** | **Maximum** | **Median (IQR)** |
| --- | --- | --- | --- |
| Pulmonary nodules | 10 | 100 | 88 (72-99) |
| Retro-orbital masses | 0 | 100 | 84 (60-96) |
| Sinus involvement | 8 | 100 | 75 (52-87) |
| Subglottic stenosis | 0 | 100 | 75 (50-88) |
| Nasal crusting | 0 | 100 | 70 (46-84) |
| Lacrimal duct involvement | 0 | 100 | 67 (38-85) |
| Endobronchial lesions | 0 | 100 | 67 (41-83) |
| Sialadenitis | 0 | 100 | 62 (29-80) |
| Parenchymal brain involvement | 0 | 100 | 50.5 (20-71) |
| Conductive hearing loss | 0 | 100 | 50 (20-75) |
| Meningitis | 0 | 100 | 47 (15-72) |
| Pulmonary infiltrates | 0 | 100 | 40 (24-52) |
| Spinal cord lesions | 0 | 96 | 35 (18-58) |
| Cranial nerve palsy | 0 | 94 | 28 (10-50) |
| Cardiomyopathy | 0 | 84 | 27 (9-50) |
| Valvular involvement | 0 | 100 | 26.5 (9-51) |
| Oral ulcers | 0 | 96 | 25 (12-48) |
| Interstitial lung disease | 0 | 85 | 24 (5-50) |
| Cutaneous ulcers | 0 | 100 | 21 (9-42) |
| Sensorineural hearing loss | 0 | 100 | 20 (5-50) |
| Uveitis | 0 | 100 | 18 (2-40) |
| Conjunctivitis | 0 | 90 | 17.5 (5-39) |
| Pericarditis | 0 | 90 | 17 (5-39) |
| Scleritis | 0 | 90 | 17 (6-49) |
| Peripheral neuropathy | 0 | 98 | 15 (2.5-23.5) |
| Constitutional symptoms | 0 | 100 | 13 (1-31) |
| Stroke | 0 | 90 | 11 (2-29) |
| Creatinine elevation | 0 | 83 | 10 (4-27) |
| Cutaneous gangrene | 0 | 100 | 10 (2-25) |
| Hematuria | 0 | 90 | 9.5 (2-23.5) |
| Mesenteric ischemia | 0 | 87 | 8 (0-24) |
| Retinal exudates | 0 | 84 | 6 (0-20) |
| Leukocytoclastic vasculitis | 0 | 98 | 6 (0-23) |
| Diffuse alveolar hemorrhage | 0 | 90 | 5.5 (0-20) |
| Proteinuria | 0 | 100 | 5 (1-24) |
| Arthritis | 0 | 71 | 3.5 (0-16) |

IQR = interquartile range

**Supplementary Table S3:** responses concerning decision of therapy for each scenario

| **Question** | **Median (IQR) response** |
| --- | --- |
| **Scenario 1:** a patient recently diagnosed with AAV who’s most severe manifestation is nodular lung disease. For your chosen co-induction agent with glucocorticoids, please indicate your level of agreement with the following statements (1 = strongly disagree, 4 = neither agree nor disagree, 7 = strongly agree) | |
| I chose this agent as it has been effective in similar patients I have treated | 6 (6-7) |
| I chose this agent as it is the local practice norm to use this agent | 6 (5-7) |
| I chose this agent as it best treats the underlying pathobiology | 6 (5-7) |
| I chose this agent because evidence suggests this agent is effective | 6 (5-7) |
| If this patient instead presented with diffuse alveolar hemorrhage as their most severe manifestation, I would use a different co-induction agent | 3 (2-6) |
| **Scenario 2:** a patient recently diagnosed with AAV who’s most severe manifestation is retro-orbital pseudotumor. For your chosen co-induction agent with glucocorticoids, please indicate your level of agreement with the following statements (1 = strongly disagree, 4 = neither agree nor disagree, 7 = strongly agree) | |
| I chose this agent as it has been effective in similar patients I have treated | 6 (6-6) |
| I chose this agent as it is the local practice norm to use this agent | 6 (4-6) |
| I chose this agent as it best treats the underlying pathobiology | 6 (5-6) |
| I chose this agent because evidence suggests this agent is effective | 5 (4-6) |
| If this patient instead presented with scleritis as their most severe manifestation, I would use a different co-induction agent | 4 (2-6) |
| **Scenario 3:** a patient recently diagnosed with AAV who’s most severe manifestation is sensorineural hearing loss. For your chosen co-induction agent with glucocorticoids, please indicate your level of agreement with the following statements (1 = strongly disagree, 4 = neither agree nor disagree, 7 = strongly agree) | |
| I chose this agent as it has been effective in similar patients I have treated | 6 (5-6) |
| I chose this agent as it is the local practice norm to use this agent | 5 (5-6) |
| I chose this agent as it best treats the underlying pathobiology | 5 (4-6) |
| I chose this agent because evidence suggests this agent is effective | 5 (4-6) |
| If this patient instead presented with sensorineural hearing loss as their most severe manifestation, I would use a different co-induction agent | 4 (2-6) |
| **Scenario 4:** a patient recently diagnosed with AAV who’s most severe manifestation is pachymeningitis. For your chosen co-induction agent with glucocorticoids, please indicate your level of agreement with the following statements (1 = strongly disagree, 4 = neither agree nor disagree, 7 = strongly agree) | |
| I chose this agent as it has been effective in similar patients I have treated | 6 (5-6) |
| I chose this agent as it is the local practice norm to use this agent | 5 (4-6) |
| I chose this agent as it best treats the underlying pathobiology | 5 (4-6) |
| I chose this agent because evidence suggests this agent is effective | 5 (4-6) |
| If this patient instead presented with mononeuritis multiplex as their most severe manifestation, I would use a different co-induction agent | 3 (2-6) |

IQR = interquartile range; AAV = ANCA-associated vasculitis
