# Supplementary material for: Clinician views concerning the prevalence and impact of granulomas on the diagnosis, management, and outcomes of ANCA-associated vasculitis
Source: Rheumatology (Oxford). Author manuscript; Available in PMC 2026 May 15. (PMC13179117; doi:10.1093/rheumatology/keaf585)
Supplement: supplement 2 [file NIHMS2174520-supplement-supplement_2.pdf]

Introduction and Consent

Principal Investigator: Dr. Michael Walsh, Division of Nephrology, St Joseph's Hospital, Hamilton
Co-Investigators: Dr. Mats Junek and Dr. Lynn Fussner

You are being invited to participate in a research study conducted by Dr. Junek as you are a clinician who treats patients with ANCA-associated vasculitis (AAV). This is a student research project conducted under the supervision of Dr. Walsh. The study will help the student learn more about the topic area and develop skills in research design, collection and analysis of data, and writing a research paper.

In order to decide whether or not you want to be a part of this research study, you should understand what is involved and the potential risks and benefits. This form gives detailed information about the research study, which will be discussed with you. Once you understand the study, you will be asked to sign this form if you wish to participate. Please take your time to make your decision. Feel free to discuss it with your friends and family, or your family physician.

Why this study is being done
AAV is histopathologically defined by the presence of granulomas and/or vasculitis, and consequential tissue damage and necrosis. It has been thought that vasculitis and granulomas contribute differently to the manifestations, natural histories, and outcomes of AAV. There are, however, no consensus definitions of what are thought to be granulomatous manifestations, nor consensus concerning how manifestations associated with granulomas may behave differently than others.

Purpose of the study
This survey seeks to inform which manifestations of AAV are thought to be attributable to vasculitis and/or granulomas and how these differences may impact clinical outcomes

Participant responsibilities
The participant will be asked to complete the following online survey without any additional follow up or questions. There are no costs. The survey has three components and is expected to take approximately 15 minutes:
1) Questions concerning beliefs around the impact of granulomas on the pathobiology, diagnosis, management, and outcomes in AAV.
2) Questions that ask to what extent a given manifestation of AAV is attributable to the formation of granulomas
3) Patient scenarios where the respondent is asked to indicate how the presence of granulomatous and/or vasculitic manifestations may impact their clinical decision-making.

Possible risks and benefits
By completing this study you will help evolve existing knowledge concerning individuals with vasculitis, their outcomes, and contribute to future research opportunities. There are no anticipated harms as no private information will be collected, however, if you have any concerns with completing the study you can stop at any time and withdraw consent (below).

Paricipation process
By clicking 'yes' to the question below, you consent to participate in this research project, having your demographic information and survey responses recorded, and the analysis and dissemination of this information in future research. Your participation is entirely voluntary and may be withdrawn at any time by closing the survey or, after completing the survey, contacting the organizers (junekm@mcmaster.ca) with the time you completed the survey. No directly identifying information will be gathered, responses will be flagged by the time of response and by a randomidentifier. You may not benefit directly from this project but will contribute to future research initaitives. Your responses will be kept completely confidential and only be available to researchers, anonymous responses may be available for review or further research upon request. If the results of the study are published, your name will not be used and no information that discloses your identity will be released or published. Please note that you must be 18 years or older to participate in this study.

Questions, concerns, withdrawal, and ethics approval
If you have any questions or concerns please contact junekm@mcmaster.ca. This study has been reviewed by the Hamilton Integrated Research Ethics Board (HIREB). The HIREB is responsible for ensuring that participants are informed of the risks associated with the research, and that participants are free to decide if participation is right for them. If you have any questions about your rights as a research participant, please call the Office of the Chair, Hamilton Integrated Research Ethics Board at 905.521.2100 x 42013 (approval 14583). For the purposes of ensuring proper monitoring of the research study, it is possible that representatives of the Hamilton Integrated Research Ethics Board, this institution, and affiliated sites may consult your original research data to check that the information collected for the study is correct and follows proper laws and guidelines.

We thank you for participating in this survey.

Do you consent to participate in this survey and have your information non-identifiably collected?

- ○ Yes
- ○ No

Have you completed this survey before?

- ○ Yes
- ○ No

Respondent Demographics

Approximately how many patients with AAV have you cared for?

- ◯ Fewer than 50 patients with vasculitis
- ◯ 50 to 99 patients with vasculitis
- ◯ 100 to 249 patients with vasculitis
- ◯ 250 to 499 patients with vasculitis
- ◯ 500 or more patients with vasculitis

How long have you been caring for patients with AAV?

- ◯ I am completing training
- ◯ Less than 5 years
- ◯ ≥5 to <10 years
- ◯ ≥10 to <15 years
- ◯ ≥15 to <20 years
- ◯ ≥20 years

How do you manage patients with AAV?

- ◯ Completely independently
- ◯ 0-25% collaboratively with another specialty
- ◯ 26-50% collaboratively with another specialty
- ◯ 51-75% collaboratively with another specialty
- ◯ 76-100% collaboratively with another specialty

Approximately how much of your time is spent performing research?

- ◯ 0-25%
- ◯ 26-50%
- ◯ 51-75%
- ◯ 76-100%

In what country do you predominantly practice medicine?

____

What is your clinical specialty?

____

General beliefs concerning granulomas in AAV

These questions seek to understand general beliefs around the impact of granulomas upon all pathobiology, diagnosis, management, and outcomes of patients with AAV.

Where both vasculitis and granulomas are present in a given organ/tissue affected by AAV, the pathobiology is driven by granulomas.

- ◯ Strongly agree
- ◯ Agree
- ◯ Somewhat agree
- ◯ Neither agree nor disagree
- ◯ Somewhat disagree
- ◯ Disagree
- ◯ Strongly disagree

The presence of symptomatic granulomas is necessary for each of the following diagnoses

	Strongly disagree	Disagree	Somewhat disagree	Neither agree nor disagree	Somewhat agree	Agree	Strongly agree
Granulomatosis with polyangiitis	◯	◯	◯	◯	◯	◯	◯
Microscopic polyangiitis	◯	◯	◯	◯	◯	◯	◯
ANCA-negative vasculitis	◯	◯	◯	◯	◯	◯	◯

Granulomatous manifestations of AAV have differential responses to therapy.

- ◯ Strongly agree
- ◯ Agree
- ◯ Somewhat agree
- ◯ Neither agree nor disagree
- ◯ Somewhat disagree
- ◯ Disagree
- ◯ Strongly disagree

In a patient with a non-granulomatous manifestation of AAV causing organ and/or life-threatening disease, the presence of other granulomatous manifestations impacts my choice of induction therapy.

- ○ Strongly agree
- ○ Agree
- ○ Somewhat agree
- ○ Neither agree nor disagree
- ○ Somewhat disagree
- ○ Disagree
- ○ Strongly disagree

In a patient with a non-granulomatous manifestation of AAV causing non-organ threatening, non-life threatening disease, the presence of other granulomatous manifestations impacts my choice of induction therapy.

- ○ Strongly agree
- ○ Agree
- ○ Somewhat agree
- ○ Neither agree nor disagree
- ○ Somewhat disagree
- ○ Disagree
- ○ Strongly disagree

When treating a patient with AAV, the presence of granulomatous manifestations impacts my choice of maintenance therapy.

- ○ Strongly agree
- ○ Agree
- ○ Somewhat agree
- ○ Neither agree nor disagree
- ○ Somewhat disagree
- ○ Disagree
- ○ Strongly disagree

When treating a patient with AAV, the presence of granulomatous manifestations impacts my duration of therapy.

- ◯ Strongly agree
- ◯ Agree
- ◯ Somewhat agree
- ◯ Neither agree nor disagree
- ◯ Somewhat disagree
- ◯ Disagree
- ◯ Strongly disagree

Patients with granulomatous manifestations of AAV are more likely to experience each of the following than patients without granulomatous manifestations:

	Strongly disagree	Disagree	Somewhat disagree	Neither agree nor disagree	Somwhat agree	Agree	Strongly agree
Relapse	◯	◯	◯	◯	◯	◯	◯
Death	◯	◯	◯	◯	◯	◯	◯
Infection	◯	◯	◯	◯	◯	◯	◯
Damage	◯	◯	◯	◯	◯	◯	◯

Patients with granulomatous manifestations of AAV require higher cumulative doses of glucocorticoids than those without granulomatous manifestations.

- ◯ Strongly agree
- ◯ Agree
- ◯ Somewhat agree
- ◯ Neither agree nor disagree
- ◯ Somewhat disagree
- ◯ Disagree
- ◯ Strongly disagree

Manifestations of ANCA vasculitis

Across all individuals with GPA and MPA, how frequently are the following manifestations caused by granuloma?

General: Arthralgias

Never caused by granulomas	Caused to granulomas in 50% of patients	Always caused by granulomas

Mucous Membrane/Eyes: Retro-orbital mass

Never caused by granulomas	Caused to granulomas in 50% of patients	Always caused by granulomas

Respiratory: Interstitial lung disease

Never caused by granulomas	Caused to granulomas in 50% of patients	Always caused by granulomas

Mucous Membrane/Eyes: Mucous ulcers

Never caused by granulomas	Caused to granulomas in 50% of patients	Always caused by granulomas

Mucous Membrane/Eyes: Scleritis/Episcleritis

Never caused by granulomas	Caused to granulomas in 50% of patients	Always caused by granulomas

Abdominal: Mesenteric ischemia

Never caused by granulomas	Caused to granulomas in 50% of patients	Always caused by granulomas

Mucous Membrane/Eyes: Conjunctivitis/Keratitis

Never caused by granulomas	Caused to granulomas in 50% of patients	Always caused by granulomas

ENT: Subglottic stensois

Never caused by granulomas	Caused to granulomas in 50% of patients	Always caused by granulomas

Neurological: Cord lesions

Never caused by granulomas	Caused to granulomas in 50% of patients	Always caused by granulomas

Manifestations of ANCA vasculitis

Across all individuals with GPA and MPA, how frequently are the following manifestations caused by granuloma?

Cardiac: Cardiomyopathy

Never caused by granulomas	Caused to granulomas in 50% of patients	Always caused by granulomas

Cutaneous: Palpable purpura

Never caused by granulomas	Caused to granulomas in 50% of patients	Always caused by granulomas

Neurological: Parenchymal brain lesions

Never caused by granulomas	Caused to granulomas in 50% of patients	Always caused by granulomas

ENT: Paranasal sinus involvement

Never caused by granulomas	Caused to granulomas in 50% of patients	Always caused by granulomas

Renal: Creatinine elevation

Never caused by granulomas	Caused to granulomas in 50% of patients	Always caused by granulomas

Mucous Membrane/Eyes: Uveitis

Never caused by granulomas	Caused to granulomas in 50% of patients	Always caused by granulomas

Neurological: Cranial nerve palsy

Never caused by granulomas	Caused to granulomas in 50% of patients	Always caused by granulomas

Renal: Hematuria

Never caused by granulomas	Caused to granulomas in 50% of patients	Always caused by granulomas

Respiratory: Nodules/Cavities

Never caused by granulomas	Caused to granulomas in 50% of patients	Always caused by granulomas

Manifestations of ANCA vasculitis

Across all individuals with GPA and MPA, how frequently are the following manifestations caused by granuloma?

Respiratory: diffuse alveolar hemorrhage

Never caused by granulomas	Caused to granulomas in 50% of patients	Always caused by granulomas

Cutaneous: Cutaneous ulcers

Never caused by granulomas	Caused to granulomas in 50% of patients	Always caused by granulomas

Cardiorespiratory: Pericarditis

Never caused by granulomas	Caused to granulomas in 50% of patients	Always caused by granulomas

Renal: Proteinuria

Never caused by granulomas	Caused to granulomas in 50% of patients	Always caused by granulomas

ENT: Conductive hearing loss

Never caused by granulomas	Caused to granulomas in 50% of patients	Always caused by granulomas

Cardiac: Valvular heart disease

Never caused by granulomas	Caused to granulomas in 50% of patients	Always caused by granulomas

Neurological: Meningitis

Never caused by granulomas	Caused to granulomas in 50% of patients	Always caused by granulomas

General: Constitutional symptoms

Never caused by granulomas	Caused to granulomas in 50% of patients	Always caused by granulomas

ENT: Nasal crusting, bloody discharge and/or ulcers

Never caused by granulomas	Caused to granulomas in 50% of patients	Always caused by granulomas

Manifestations of ANCA vasculitis

Across all individuals with GPA and MPA, how frequently are the following manifestations caused by granuloma?

Respiratory: Endobronchial involvement

Never caused by granulomas	Caused to granulomas in 50% of patients	Always caused by granulomas

Cutaneous: Cutaneous infarcts/gangrene

Never caused by granulomas	Caused to granulomas in 50% of patients	Always caused by granulomas

ENT: Sensorineural hearing loss

Never caused by granulomas	Caused to granulomas in 50% of patients	Always caused by granulomas

ENT: Salivary gland swelling

Never caused by granulomas	Caused to granulomas in 50% of patients	Always caused by granulomas

Neurological: Peripheral neuropathy

Never caused by granulomas	Caused to granulomas in 50% of patients	Always caused by granulomas

Mucous Membrane/Eyes: Retinal exudates/hemorrhage

Never caused by granulomas	Caused to granulomas in 50% of patients	Always caused by granulomas

Neurological: Stroke

Never caused by granulomas	Caused to granulomas in 50% of patients	Always caused by granulomas

Respiratory: Infiltrates

Never caused by granulomas	Caused to granulomas in 50% of patients	Always caused by granulomas

Mucous Membrane/Eyes: Lacrimal duct/gland involvement

Never caused by granulomas	Caused to granulomas in 50% of patients	Always caused by granulomas

Patient Decision Scenario

You are seeing a patient in your clinic who was recently diagnosed with severe AAV. Their most severe manifestation at presentation is nodular lung disease. They were started on oral glucocorticoids by the referring provider and are current vitally stable and appear well. Please answer the following questions based on this prompt:

What agent would you use as induction co-therapy with glucocorticoids?

[ ]

I chose this agent for the following reasons:

	Strongly disagree	Disagree	Somewhat disagree	Neither agree nor disagree	Somewhat agree	Agree	Strongly agree
It has been effective in similar patients I have treated	○	○	○	○	○	○	○
It is the local practice norm to use this agent	○	○	○	○	○	○	○
This agent best treats the underlying pathobiology of the disease	○	○	○	○	○	○	○
Evidence suggests that this is the most efffective therapy for this manifestation	○	○	○	○	○	○	○

If the same patient instead presented to me with diffuse alveolar hemorrhage as their most severe manifestation, I would use a different induction agent to administer as co-therapy with glucocorticoids.

- ○ Strongly agree
- ○ Agree
- ○ Somewhat agree
- ○ Neither agree nor disagree
- ○ Somewhat disagree
- ○ Disagree
- ○ Strongly disagree

If you would use a different agent, what would be your alternative choice of co-induction agent? Leave the box blank if you would not change agents.

[ ]

Patient Decision Scenario

You are seeing a patient in your clinic who was recently diagnosed with severe ANCA-associated vasculitis. Their most severe manifestation at presentation was retro-orbital pseudotumor. They were started on oral glucocorticoids by the referring provider and are current vitally stable and appear well. Please answer the following questions and agree/disagree with the following statements based on this prompt:

What agent would you use as induction co-therapy with glucocorticoids?

[ ]

I chose this co-induction agent for the following reasons:

	Strongly disagree	Disagree	Somewhat disagree	Neither agree nor disagree	Somewhat agree	Agree	Strongly agree
It has been effective in similar patients I have treated	○	○	○	○	○	○	○
It is the local practice norm to use this agent	○	○	○	○	○	○	○
This agent best treats the underlying pathobiology of the disease	○	○	○	○	○	○	○
Evidence suggests that this is the most efffective therapy for this manifestation	○	○	○	○	○	○	○

If the same patient instead presented to me with scleritis as their most severe manifestation, I would use a different induction agent to administer as co-therapy with glucocorticoids.

- ○ Strongly agree
- ○ Agree
- ○ Somewhat agree
- ○ Neither agree nor disagree
- ○ Somewhat disagree
- ○ Disagree
- ○ Strongly disagree

If you would use a different agent, what would be your alternative choice of co-induction agent? Leave the box blank if you would not change agents.

[ ]

Patient Decision Scenario

You are seeing a patient in your clinic who was recently diagnosed with severe ANCA-associated vasculitis. Their most severe manifestation at presentation was conductive hearing loss. They were started on oral glucocorticoids by the referring provider and are current vitally stable and appear well. Please answer the following questions and agree/disagree with the following statements based on this prompt:

What agent would you use as induction co-therapy with glucocorticoids?

[ ]

I chose this co-induction agent for the following reasons:

	Strongly disagree	Disagree	Somewhat disagree	Neither agree nor disagree	Somewhat agree	Agree	Strongly agree
It has been effective in similar patients I have treated	○	○	○	○	○	○	○
It is the local practice norm to use this agent	○	○	○	○	○	○	○
This agent best treats the underlying pathobiology of the disease	○	○	○	○	○	○	○
Evidence suggests that this is the most efffective therapy for this manifestation	○	○	○	○	○	○	○

If the same patient instead presented to me with sensorineural hearing loss as their most severe manifestation, I would use a different induction agent to administer as co-therapy with glucocorticoids.

- ○ Strongly agree
- ○ Agree
- ○ Somewhat agree
- ○ Neither agree nor disagree
- ○ Somewhat disagree
- ○ Disagree
- ○ Strongly disagree

If you would use a different agent, what would be your alternative choice of co-induction agent? Leave the box blank if you would not change agents.

[ ]

Patient Decision Scenario

You are seeing a patient in your clinic who was recently diagnosed with severe ANCA-associated vasculitis. Their most severe manifestation at presentation was pachymeningitis. They were started on oral glucocorticoids by the referring provider and are current vitally stable and appear well. Please answer the following questions and agree/disagree with the following statements based on this prompt:

What agent would you use as induction co-therapy with glucocorticoids?

[ ]

I chose this co-induction agent for the following reasons:

	Strongly disagree	Disagree	Somewhat disagree	Neither agree nor disagree	Somewhat agree	Agree	Strongly agree
It has been effective in similar patients I have treated	○	○	○	○	○	○	○
It is the local practice norm to use this agent	○	○	○	○	○	○	○
This agent best treats the underlying pathobiology of the disease	○	○	○	○	○	○	○
Evidence suggests that this is the most efffective therapy for this manifestation	○	○	○	○	○	○	○

If the same patient instead presented to me with mononeuritis multiplex as their most severe manifestation, I would use a different induction agent to administer as co-therapy with glucocorticoids.

- ○ Strongly agree
- ○ Agree
- ○ Somewhat agree
- ○ Neither agree nor disagree
- ○ Somewhat disagree
- ○ Disagree
- ○ Strongly disagree

If you would use a different agent, what would be your alternative choice of co-induction agent? Leave the box blank if you would not change agents.

[ ]
